# Fixed Full Arches Supported by Tapered Implants with Knife-Edge Thread Design and Nanostructured, Calcium-Incorporated Surface: A Short-Term Prospective Clinical Study

**DOI:** 10.1155/2017/4170537

**Published:** 2017-01-29

**Authors:** Soheil Bechara, Algirdas Lukosiunas, Giorgio Andrea Dolcini, Ricardas Kubilius

**Affiliations:** ^1^Private Practice, MIR International Implantology Center, LT-08339 Vilnius, Lithuania; ^2^Department of Oral and Maxillofacial Surgery, Lithuanian University of Health Science, LT-44307 Kaunas, Lithuania; ^3^Private Practice, 21100 Como, Italy

## Abstract

*Purpose.* To evaluate implant survival, peri-implant bone loss, and complications affecting fixed full-arch (FFA) restorations supported by implants with a knife-edge thread design and nanostructured, calcium-incorporated surface.* Methods.* Between January 2013 and December 2015, all patients referred for implant-supported FFA restorations were considered for enrollment in this study. All patients received implants with a knife-edge thread design and nanostructured calcium-incorporated surface (Anyridge®, Megagen, South Korea) were restored with FFA restorations and enrolled in a recall program. The final outcomes were implant survival, peri-implant bone loss, biologic/prosthetic complications, and “complication-free” survival of restorations.* Results.* Twenty-four patients were selected. Overall, 215 implants were inserted (130 maxilla, 85 mandible), 144 in extraction sockets and 71 in healed ridges. Thirty-six FFAs were delivered (21 maxilla, 15 mandible): 27 were immediately loaded and 9 were conventionally loaded. The follow-up ranged from 1 to 3 years. Two fixtures failed, yielding an implant survival rate of 95.9% (patient-based). A few complications were registered, for a “complication-free” survival of restorations of 88.9%.* Conclusions.* FFA restorations supported by implants with a knife-edge thread design and nanostructured, calcium-incorporated surface are successful in the short term, with high survival and low complication rates; long-term studies are needed to confirm these outcomes.

## 1. Introduction

Dental implants are a long-term reliable solution for the prosthetic rehabilitation of partially and totally edentulous patients, with high rates of survival and success in the short, medium, and long term [1–3]. In particular, in fully edentulous patients, rehabilitation with implant-supported prostheses is a solution that can effectively restore chewing function and aesthetics, resulting in significant improvement in quality of life, both personally and socially [4, 5].

Several studies have reported high survival and success rates between 95% and 100% for the rehabilitation of totally edentulous jaws with fixed full-arch (FFA) prostheses [6–14]. Most of these studies, however, took into account mandibular FFA prostheses [6–10]; fewer studies have reported the results of maxillary FFA rehabilitations [11–14], especially when supported by immediately loaded implants [11–13] or immediate postextraction implants [14].

It has long been known that along with the general medical condition of the patient, type of fixture used, and surgical and prosthetic protocols, the quality and quantity of bone are key factors in determining implant survival and the success of osseointegration [15, 16]. Adequate primary implant stabilization during the surgical act, of a mechanical nature, is essential for the successful osseointegration and deposition of new bone on the implant surface, during the first period of healing: the initial primary stabilization must, in fact, be replaced by an adequate secondary, biological stabilization [16, 17]. As a consequence, it is clear that placement of an implant-supported FFA prosthesis in the mandible may be characterized by a lower risk of failure, compared with that in the maxilla. In fact, mandibular bone quality is higher, facilitating the primary stabilization of implants [6, 7, 11, 12, 14, 18].

It is also evident that the placement of implants in completely healed edentulous ridges with submerged healing and delayed loading may involve lower risks than insertion of the fixtures in extraction sockets with immediate functional loading [14, 19, 20].

The requirements of modern implantology, however, stimulate the surgeon and prosthodontist to speed up the implant/prosthetic treatment. In fact, patients ask that their lost or compromised teeth be replaced as soon as possible, without the interim period of removable full dentures [11, 16, 20, 21].

To meet these demands, implant systems with special features have been introduced into the market: new implant designs (macrotopographies) that maximize the primary stabilization in difficult contexts (e.g., sites with poor bone density, like the posterior maxilla, or postextraction sockets) have been proposed [21–23], as well as new implant surfaces (micro- or even nanotopographies) able to accelerate deposition of new bone onto the fixture and, therefore, secondary stabilization [15, 17, 24, 25].

The aim of the study described here was to evaluate FFA restorations supported by tapered implants with a peculiar macrothread design for enhanced primary stabilization and a novel nanostructured, calcium-incorporated surface for secondary stabilization. Outcomes assessed included implant survival, peri-implant bone resorption, and biological and prosthetic complications that affected the implant-supported rehabilitation, over the entire observation period.

## 2. Materials and Methods

### 2.1. Patient Selection

In the period January 2013 to December 2015, all patients referred to two dental clinics in Kaunas, Lithuania (specifically, a private dental clinic and the oral surgery unit of the University Hospital), for treatment with dental implants, were considered for enrollment in this prospective clinical study. Patients who had a totally edentulous maxilla and/or mandible, were wearing completely removable dentures, and wanted to restore their chewing function and aesthetics through an implant-supported, FFA rehabilitation and patients who required multiple extractions in the maxilla and/or mandible and replacement of compromised elements with implant-supported FFA prostheses were included in this study. All patients had to be in good general and oral health and had to be able to understand and sign an informed consent form. The presence of dentition in the opposing jaw (natural teeth, tooth-borne or implant-supported fixed partial prosthesis, and complete denture) was also a condition for the inclusion in the present study, where the rehabilitation of both arches with FFA prostheses was not needed. Excluded from this study were patients with severe systemic disease that represented a serious contraindication to surgery; immunocompromised patients; patients with uncompensated/uncontrolled diabetes; patients taking anticoagulants; patients undergoing radio- or chemotherapy of the head or neck; patients being treated with oral and/or intravenous aminobisphosphonates; and patients with psychiatric and/or psychological disorders. Cigarette smoking was not in itself a reason for exclusion from this study; however, patients were notified of the increased possibility of implant therapy failure related to this risk factor [26]. Moreover, a history of bruxism and/or parafunction did not represent an exclusion criteria in the present study. Each patient was informed in detail of the surgical and prosthetic protocols of the study and signed an informed consent to treatment. This clinical study was carried out in accordance with the principles set out in the Helsinki Declaration on clinical trials involving human subjects (2008 review).

### 2.2. Preoperative Clinical and Radiographic Evaluation

Placement of the implant was preceded by a detailed clinical and radiological analysis. Clinical analysis consisted of a visit during which impressions were obtained and plaster models developed for diagnostic wax-up, to determine the specific clinical needs of each patient. The preliminary radiographic analysis consisted of two-dimensional panoramic radiography for general evaluation of the amount of bone available; such an investigation could be supplemented, when necessary, with a three-dimensional (3D) evaluation with cone beam computed tomography (CBCT). The data derived from CBCT were exported as DICOM (Digital Imaging and Communication in Medicine) files and loaded into a suitable 3D reconstruction software (R2Gate®, Megagen), with which the surgeon could make a 3D reconstruction of the bony architecture and obtain all possible information related to the quantity (height and thickness) and quality (density) of the residual bone.

### 2.3. Implant Macro-, Micro-, and Nanotopography

The implants used in this study (AnyRidge, Megagen) were tapered implants, characterized by a peculiar knife-edge thread design (Knifethread®) that ensures a high primary stabilization, even in difficult clinical contexts, as in the case of immediate loading or in sites characterized by a small amount (e.g., postextraction sockets) or low quality (e.g., posterior maxilla) of bone [21–23]. On the microsurface topography determined by a sandblasting treatment (resorbable blast media) was superimposed a nanotopography determined by incorporation of calcium ions (Xpeed®, Megagen), to increase energy and surface area, with the aim of strengthening and accelerating osseointegration [24]. From the prosthetic point of view, such implants possessed a 5 mm deep conical connection (10°) combined with an internal hexagon, ensuring high mechanical stability and a suitable biological seal; an integrated switching platform was present, to maintain peri-implant tissue volume over time [27]. The implants were available in various lengths (7, 8.5, 10, 11.5, and 13 mm) and diameters (3.5, 4, 4.5, and 5 mm) depending on the surgeon's needs.

### 2.4. Surgical Protocol

Patients attended a professional hygiene session 1 wk prior to the intervention and received a prescription for a mouth rinse containing chlorhexidine 0.12%, to be used two to three times daily for the 5 days prior to surgery.

All surgical procedures were carried out by the same surgeon with experience in the field of implantology (S.B.); in all cases, a one-stage surgical technique was selected, with no submerged healing. Anesthesia was provided by infiltration of articaine 4% with epinephrine (1 : 100,000).

In completely edentulous patients, the surgeon made a wide crestal incision, connected to two vertical releasing incisions. After elevating a full-thickness flap, he started the osteotomy under copious saline irrigation, with a 2.0 mm drill. Then he proceeded through a sequence of drills of increasing diameter, as indicated by the implant manufacturer. The clinician's decision to stop depended on the anatomical situation (available bone quantity/quality).

For patients who required multiple extractions, removal of all teeth, and placement of immediate postextraction implants, the surgeon opted for a flapless approach. Compromised teeth were gently extracted. Extraction sockets were carefully cleaned to remove any remaining infected or granulation tissue. After carefully checking the integrity of extraction socket walls with the periodontal probe, the surgeon proceeded to preparation of the implant site using, first, a 2.0 mm drill, under copious saline irrigation. As a general rule, the preparation was carried out 3-4 mm beyond the apex of the socket, and the sites were underprepared, to ensure better stabilization of the implant. Therefore, guided by his experience and the quality of bone present, the surgeon proceeded in site preparation and might use only the first preparatory drills and not those of larger diameter.

**Figure 1:**
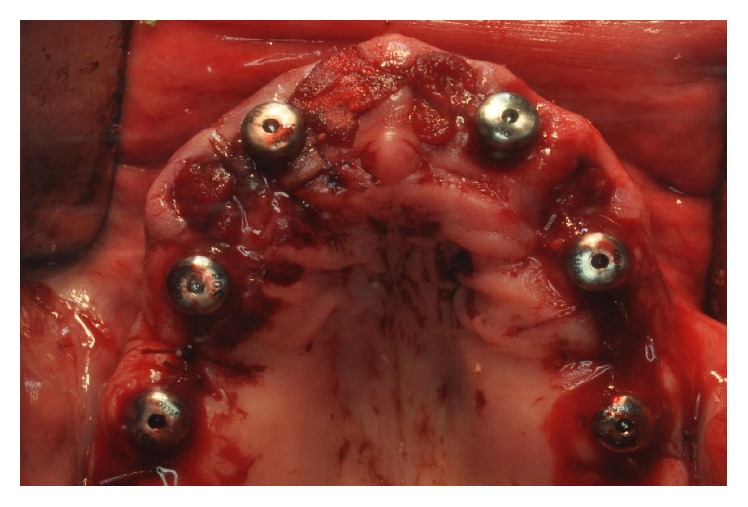
Implant placement in the maxilla. The anterior implants were placed in postextraction sockets, immediately after extractions; the posterior implants were placed in healed ridges. In total, six implants were placed to support a maxillary FFA restoration.

**Figure 2:**
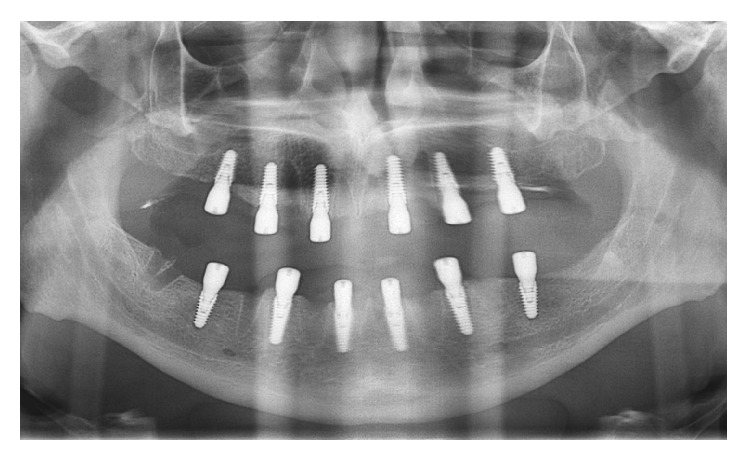
Panoramic radiograph taken immediately after implant placement.

The surgeon was free to choose between different lengths and diameters depending on clinical needs. Implants were placed with the surgical motor, which was used to accurately record the insertion torque. If the final torque of implant placement was <40 N·cm, the fixtures were not considered candidates for immediate loading; at torques ≥40 N·cm, the implants were eligible for immediate loading. Where the insertion torque was >50 N·cm, the surgeon stopped the surgical motor and completed implant insertion manually, with a hand ratchet. In each patient, the surgeon could insert four to eight implants depending on the predetermined treatment plan (Figures 1 and 2).

After placing the implants in healed sites, the surgeon could choose to strengthen and protect the buccal cortical bone with autogenous bone particles (recovered during preparation of the implant site) mixed with xenograft material; in all postextraction sites, the gap between the coronal part of the implant and the residual alveolus walls was filled with the aforementioned mixture of autogenous bone particulate and xenograft granules.

Finally, the healing abutments were positioned. In patients with immediate postextraction implants, larger-diameter abutments were chosen to protect the socket, which was filled with regenerative material; in most cases, suturing was not necessary. In patients with implants inserted in healed ridges, however, the mucoperiosteal full-thickness flap was sutured with interrupted sutures.

Patients who were already completely edentulous had their complete removable dentures abundantly discharged at the healing abutment sites; patients treated with immediate postextraction implants were provided with a new interim completely removable denture, suitably relined with soft resin, and also discharged in correspondence of the healing abutments. It was recommended that patients wear their removable dentures only for aesthetics in the first days after surgery, to promote soft tissue healing. Finally, patients were prescribed antibiotics (amoxicillin + clavulanic acid, 2 g daily for 6 days in total) and analgesics (ibuprofen 600 mg, twice daily for a maximum of 3 days). Mouth rinses containing chlorhexidine 0.12%, were also prescribed (two or three times per day) for the 5-day period following surgery. The first follow-up visit was scheduled 48 hours after surgery.

### 2.5. Prosthetic Protocol

**Figure 3:**
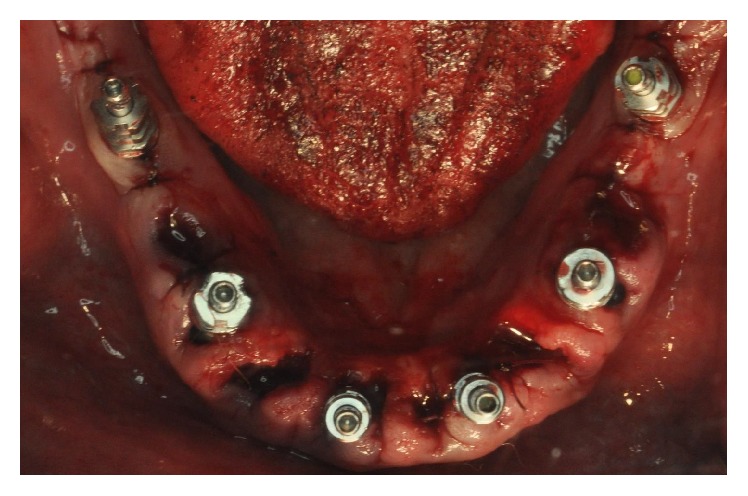
Six implants were placed in the mandible. The day after surgery impressions were taken, in order to provide patients with temporary acrylic resin FFA prostheses and to functionally load the fixtures according to an immediate loading protocol.

**Figure 4:**
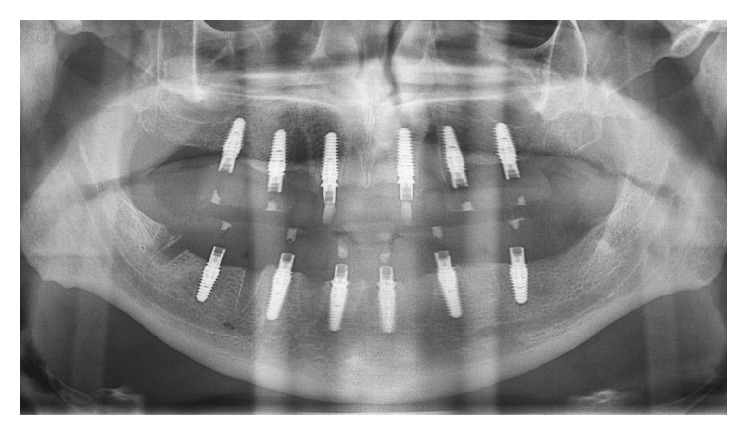
Panoramic radiograph taken 72 hours after surgery, when the implants were functionally loaded with the temporary acrylic resin FFA prostheses.

**Figure 5:**
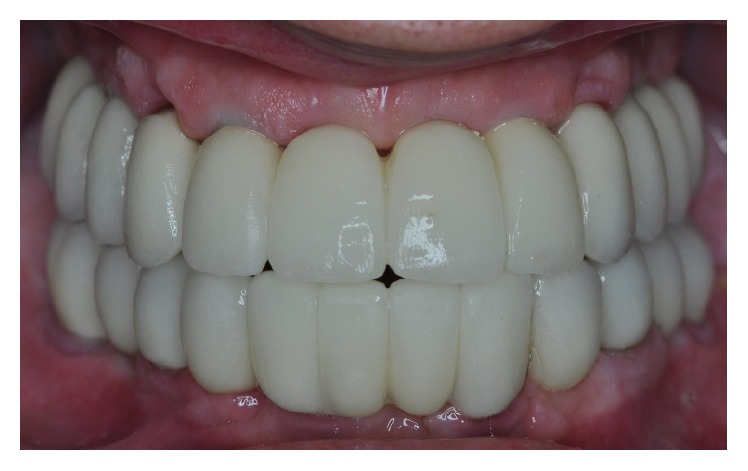
Clinical picture of the temporary acrylic resin FFAs, 1 week after surgery.

**Figure 6:**
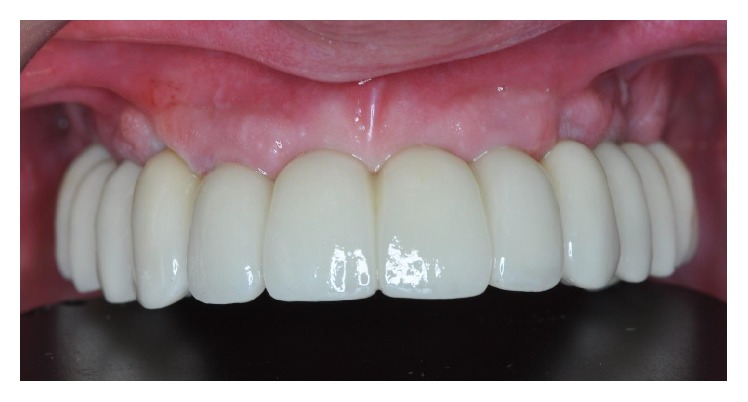
Delivery of the final metal-ceramic maxillary FFA.

For all patients in whom the final insertion torque of the majority of implants (>50%) were ≥40 N·cm, pickup impressions were made within 2 days after surgery to proceed with the immediate functionalization of the implants (Figure 3). The day after impressions were obtained, these patients were provided with a temporary FFA prosthesis, and their implants were functionally loaded according to an immediate loading protocol (Figures 4 and 5). The healing abutments were replaced with prosthetic abutments, which were screwed on the implants; a temporary FFA prosthesis in reinforced acrylic resin was positioned to fit properly and adapt to the peri-implant tissues. In the case of extraction sockets, the morphology of the temporary prosthesis allowed proper sealing of the open spaces undergoing healing. Finally, occlusion was carefully checked. The temporary restoration was cemented over the abutments and remained in place for 2 months, after which it was replaced with the definitive metal-ceramic or zirconia-ceramic FFA restoration (Figure 6).

When a majority of the fixtures being placed (>50%) had a final insertion torque <40 N·cm, a conventional loading protocol was chosen, in which a 2- to 3-month period preceded functional loading of the implants. This meant that the patient had to wear complete interim removable dentures, discharged at the healing abutment sites, for a minimum of 2 months. At the end of this period, a pickup impression was obtained to proceed with the functional loading of the implants, with a temporary acrylic reinforced resin FFA prosthesis, and this temporary restoration was replaced by the definitive one after a 2-month period, as reported above. The definitive prostheses were FFA metal-ceramic or zirconia-ceramic restorations, supported, respectively, by 4, 6, 7, or 8 implants and cemented over them.

All patients were included in a recall and follow-up control program, for which they had to be at the dental office or clinic at least two times a year, respectively, 6 and 12 months after surgery in the first year. During each scheduled follow-up visit, the patient attended a 30-minute oral hygiene session, with motivation provided by a dental hygienist; in addition, the clinician clinically monitored the patient carefully, by inspection, periodontal probing, and panoramic radiography, to detect any biological or prosthetic complication.

### 2.6. Outcomes of the Study

#### 2.6.1. Implant Survival

An implant was classified as surviving if present in the mouth and functioning at each follow-up visit. Conversely, an implant was classified as failed if lost for various reasons (mobility caused by lack of osseointegration, infection, progressive bone loss in the absence of infection, and fracture of the implant) in the first period of healing or after prosthetic loading.

#### 2.6.2. Biological Complications

Among the biological complications were inflammatory/infectious complications, such as peri-implant mucositis and peri-implantitis, but also progressive marginal bone loss in the absence of infection. Peri-implant mucositis was indicated by the presence of bleeding on probing and/or suppuration, associated with a probing pocket depth ≥4 mm, but in the absence of peri-implant bone loss [28]. Peri-implantitis was diagnosed on the basis of deep discomfort/pain, probing pocket depth ≥4 mm, bleeding on probing, and/or secretion of pus associated with peri-implant bone resorption (≥2.5 mm) [28]. Finally, in the presence of a progressive bone resorption (≥1.5 mm) without any symptom/sign of infection, progressive marginal bone loss resulting from prosthetic overload was diagnosed. The stability of peri-implant tissues was evaluated on panoramic radiographs, as previously described [23]. In brief, different panoramic radiographs were taken for each patient, at different times (immediately after implant placement, on delivery of the final FFA, and 1 and 2 years later) [23]. Radiographs were scanned, converted to TIFF (600 dpi), and saved in dedicated folders. Peri-implant bone levels were then measured with the aid of dedicated software (Scion Image®, Scion, Frederick, MD, USA). The mesial and distal bone levels of each implant were measured: reference points for linear measurements were the implant shoulder and the most coronal bone-to-implant contact [23]. To correct any distortion, the software was calibrated using the distance (known) between two consecutive threads. Then, the marginal bone resorption was calculated as the change in peri-implant marginal bone levels during the observation period; the final value was calculated as the mean of the modifications in the mesial and distal portions [23].

#### 2.6.3. Prosthetic Complications

Prosthetic complications were divided into two categories: mechanical complications were defined as all problems occurring at preformed components or at the level of the implant-abutment connection (loosening or fracture of the connecting screw, abutment fracture), whereas technical complications were described as all problems occurring at the prosthetic superstructures (loosening or fracture of provisional, debonding, chipping and/or fracture of ceramics, and fracture of the metal framework) [3, 10, 29]. Mechanical and technical complications were described as minor complications if they required less than 30 minutes chairside for resolution; conversely, they were described as major if they required more than 30 minutes of chairside or the intervention of a dental technician for resolution.

#### 2.6.4. “Complication-Free” Survival of Restorations

Only prostheses that did not exhibit any problem (no implant loss and no biological or prosthetic complications throughout the entire observation period of the study) could be defined as “complication-free” [3, 10]. If even a single adverse event occurred (to a single implant), the prosthesis could not be described as “complication-free” and therefore was categorized in the group of prostheses with complications [3, 10].

### 2.7. Statistical Analysis

The distributions of patients and implants were studied by means of descriptive statistical analysis. In particular, for quantitative variables (patient age, peri-implant bone resorption) mean, standard deviation, median, range, and 95% confidence intervals (CIs) were calculated; for patient-related qualitative variables (gender, age, smoking history, and periodontal disease history), implant-related variables (site, location, surgical protocol, quality and bone conditions, insertion torque, and implant length and diameter), and prosthesis-related variables (site, opposing dentition, loading protocol, and number of supporting implants), absolute (expressed as a number, *n*), and relative (expressed as a percentage) frequency distributions were calculated. *χ*^2^ test was used to calculate the differences in distribution between the groups, with the significance level set at 0.005. Finally, implant survival, incidence of biological and prosthetic complications, and “complication-free” survival of prostheses were calculated at the patient, implant, and restoration levels, respectively. In calculations at the patient and restoration levels, even a single adverse event (failure or complication) automatically classified the patient or restoration as a “failure” or “complication.” All calculations were made with the aid of a statistical spreadsheet (Excel 2003®, Microsoft, Redmond, WA, USA).

## 3. Results

### 3.1. Distribution of Patients, Implants, and Restorations

Twenty-four patients (4 men and 20 women) aged between 34 and 77 years (mean age, 54.9 ± 14.4 years; median, 53 years; 95% CI, 49.2–60.6 years) were enrolled in this prospective clinical study between January 2013 and December 2015. With respect to the distribution of patients, more women were enrolled (*p* = 0.001) and most patients were nonsmoking (*p* = 0.004), although the percentage of smokers was rather high (5/24, 20.8%). Finally, all patients (*p* < 0.0001) had history of periodontal disease (24/24, 100%), which had resulted in loss of all teeth or severe impairment of remaining teeth, which were severely compromised and not savable.  Table 1 summarizes all patient-related information.

Two hundred and fifteen implants were inserted, 130 in the maxilla (130/215, 60.5%) and 85 in the mandible (85/215, 39.5%). Given that most of the fixtures were positioned in the upper arch, the two groups differed statistically (*p* = 0.002) in distribution. Forty-five implants were placed in the anterior maxilla (45/215, 20.9%), and 85 were placed in the posterior maxilla (85/215, 39.5%); 32 implants were placed in the anterior mandible (32/215, 14.9%), and 53 implants were located in the posterior mandible (53/215, 24.7%). Again, the groups differed statistically in distribution (*p* < 0.0001). More than half of the implants were in fact inserted in posterior regions (138/215, 64.2%), and fewer fixtures (77/215, 35.8%) were placed in anterior regions.

With respect to the surgical protocol, more than half of the fixtures were placed in postextraction sockets (144/215. 67%); only 71 implants were placed in fully healed edentulous ridges (71/215. 33%), resulting in a statistically significant difference in the distribution between these groups (*p* < 0.0001), as was the case for bone quality. In fact, most of the implants were inserted into type III bone (143/215, 66.5%) and type IV bone (58/215, 27%); only a few implants were placed in type I or II bone (14/215, 6.5%), that is, bone of superior quality. Around 64 implants (64/215, 29.8%) were placed in native bone, whereas 151 of 215 implants (70.2%) were inserted simultaneously with bone regeneration or in previously regenerated bone. Because, by protocol, all postextraction implants (144) were placed with simultaneous preservation of the socket (i.e., filling of the socket with autologous bone mixed with xenograft granules), only 7 implants were placed in regenerated sites (sites that were previously regenerated with the aim of increasing the height and thickness of the residual alveolar ridge). The technique used in this case was the sinus lift.

With respect to insertion torque, the vast majority of the implants were placed with a final insertion torque ≥40 N·cm (192/215, 89.3%); for only 23 fixtures (23/215, 10.7%) the insertion torque was <40 N·cm. The distribution was statistically nonhomogeneous (*p* < 0.0001). The distribution of implants was not homogeneous for length (*p* < 0.0001), with a prevalence of 11.5 mm (73/215, 33.9%), 10 mm (52/215, 24.2%), and 13 mm (44/215, 20.5%) implants. Similarly, the distribution was not homogeneous with respect to diameter (*p* < 0.0001), with a clear predominance of large 4 mm (92/215, 42.8%) and 3.5 mm (88/215, 40.9%) implants compared with fixtures of larger diameter.  Table 2 summarizes all implant-related information.

There were a total of 36 FFAs. Twenty-one FFAs were in the maxilla and 15 in the mandible: although the maxilla was the most frequent site, the difference was not significant (*p* = 0.317). Twelve patients required FFA rehabilitation in both arches, and another 12 required an FFA rehabilitation in only one arch. Among the latter, only 2 patients had a completely removable denture with resin teeth as antagonist; conversely, 10 patients had natural dentition in the opposing arch.

The distribution of FFAs according to opposing dentition was statistically inhomogeneous (*p* < 0.0001). With respect to the loading protocol, the majority of FFAs (27/36, 75%) were loaded immediately, whereas only 9 of 36 (25%) were conventionally loaded, with 2-3 months of healing before loading. The distribution of implants according to type of prosthetic loading was therefore statistically inhomogeneous (*p* = 0.002). Finally, 8 FFAs were supported by 4 implants (8/236, 22.2%), 20 FFAs were supported by 6 implants (20/36, 55.6%), 1 FFA was supported by 7 implants (1/36, 2.8%), and 7 FFAs were supported by 8 implants (7/36, 19.4%). Once again, the distribution was statistically inhomogeneous (*p* = 0.0001). All restoration-related information is provided in Table 3.

### 3.2. Implant Survival

**Figure 7:**
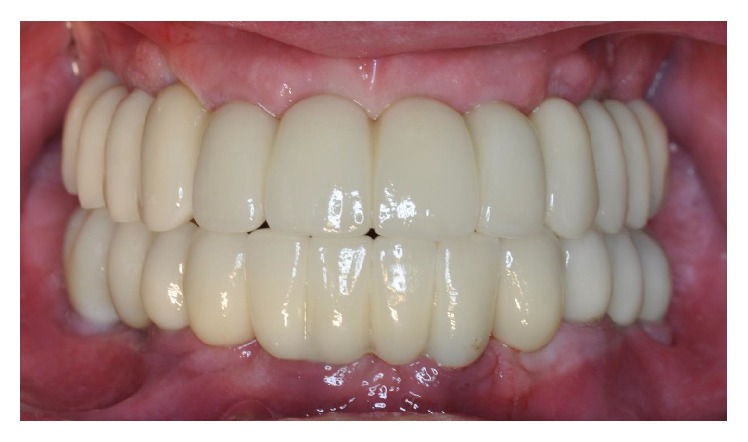
The final metal-ceramic FFAs at the 3-year follow-up control.

Follow-up ranged from 1 to 3 years (mean follow-up, 2.0 ± 0.8 years; median, 2 years, 95% CI, 1.74–2.2 years). All patients responded to the surveys, and there were no dropouts. There were only 2 failures, and these occurred in a 67-year-old, nonsmoking male patient; in this patient, the implants were placed according to the conventional surgical protocol, in the posterior maxilla, in the first and second molar positions. These failures occurred before loading (and were therefore defined as early failures) because of nonintegration and lack of stability of the fixtures, caused by failure of the sinus lift regenerative procedure; these implants were replaced after a second sinus lift, and no further failures occurred. The incidence of failures was therefore 4.1% (patient-based, 1/24 patients) and 0.9% (implant-based, 2/215 implants), respectively. In the present study, we did not register failures for infection or for progressive marginal bone loss caused by mechanical overload. No implant fracture occurred. At the end of the study, implant survival was 95.9% (patient-based) and 99.1% (implant-based), respectively (Figure 7).

### 3.3. Biological Complications

**Figure 8:**
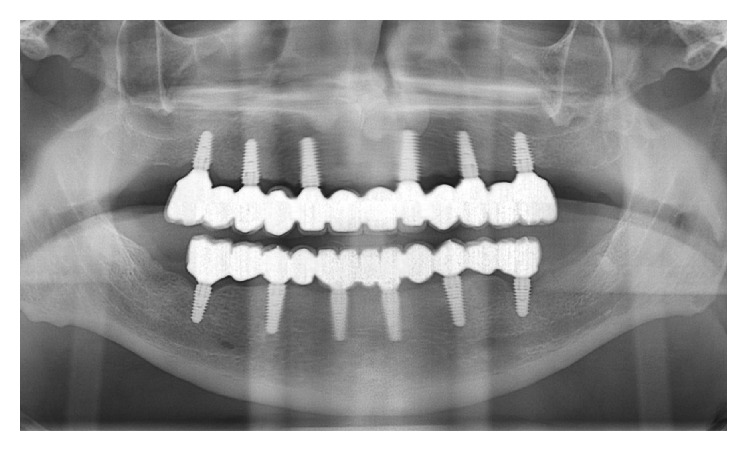
Panoramic radiograph of the FFAs at the 3-year follow-up control.

In a 47-year-old smoking patient, 2 years after placement of the implants, a transient inflammation of the marginal areas, with bleeding on probing (but without marginal bone loss), occurred. This adverse event, defined as peri-implant mucositis, involved two fixtures; although this situation was resolved through professional oral hygiene sessions, it represented a biological complication. The incidence of biological complications was 4.1% (patient-based, 1/24 patients), 0.9% (implant-based, systems 2/215), and 2.7% (restoration-based, 1/36 FFAs), respectively. No other biological complications (such as peri-implantitis and progressive marginal bone loss in the absence of infection) occurred. As reported in Table 4, the average peri-implant bone resorption values were 0.21 ± 0.15 mm (median, 0.18 mm; 95% CI, 0.19–0.23 mm) and 0.25 ± 0.11 mm (median, 0.25 mm; 95% CI, 0.24–0.26 mm), respectively, 1 and 3 years after implant placement (Figure 8).

### 3.4. Prosthetic Complications

Six prosthetic complications occurred: 4 affected the temporary FFAs (fractures of provisional acrylic resin restorations, which were repaired or replaced), and only 2 affected the definitive FFAs (chipping and/or fracture of the ceramic). All these complications were technical in nature and were classified as major complications, because they required more than 30 minutes of chair time for repair, as well as the technician's intervention. In particular, the definitive FFAs subject to chipping and fracture of ceramics had to be removed and repaired. The incidence of prosthetic complications affecting definitive FFAs was thus 5.5% (2/36 FFAs).

### 3.5. “Complication-Free” Survival of Restorations

Four FFAs failed and/or had complications. In fact, 2 implant failures, 1 biological complication (although mild and fully reversible, such as peri-implant mucositis), and 2 technical complications (chipping and/or fracture of ceramics: major complications that required repair by the dental technician) occurred in 4 different patients. Therefore, the overall incidence of failures and complications of the final fixed restorations was 11.1% (restoration-based: 4/36 FFAs), yielding a “complication-free” survival rate for restorations of 88.9%.

## 4. Discussion

To date, several studies have analyzed the survival and success of implant-supported FFA rehabilitations [6–14], particularly in the lower jaw [6–10]. However, few studies have addressed the subject of implant-supported maxillary FFA rehabilitations [10–14], particularly with implants placed in fresh extraction sites [14] or immediately loaded [12, 13].

In a nonrandomized controlled study, Peñarrocha-Oltra and colleagues [11] compared patient satisfaction and postoperative discomfort for immediate versus conventional loading in partially edentulous patients requiring extraction of the remaining maxillary dentition and rehabilitation with FFA prostheses. Thirty patients scheduled for FFA implant-supported maxillary rehabilitation were enrolled in this study: 15 were treated with conventional loading and the next 15 with immediate loading [11]. Postoperative discomfort was assessed immediately after surgery; patient satisfaction, comprising several different parameters (function, esthetics, speaking, comfort, self-esteem, ease of cleaning, and treatment duration), was assessed preoperatively and 3 and 12 months postoperatively [11]. This study revealed that patient satisfaction with immediate loading was significantly higher than that with conventional loading during the osseointegration period; after 12 months, however, when final FFAs had been functioning for some time, this difference disappeared [11]. No differences were found between loading protocols with respect to postoperative discomfort and swelling [11].

In another nonrandomized controlled study, the same authors compared immediate and conventional loading of FFA maxillary prostheses supported by implants placed in healed and fresh postextraction sockets [14]. Thirty patients requiring FFA maxillary prostheses supported by implants placed in healed and fresh extraction sites were selected for this study: 15 patients were treated with conventional loading (control group) and 15 were treated with immediate loading (test group) [14]. Each patient received 6 to 8 implants; fixtures with insertion torques <35 N·cm were conventionally loaded. The outcomes of the study were implant success, biological and prosthetic complications, success of the immediately loaded provisional prostheses, and marginal bone loss [14]. At the end of the study, the sample included 29 patients and 193 implants (94 test implants, 99 control implants). Implant success rates were 96.8% (test) and 99.0% (control) [14]. In the test group, the most common complications were abutment screw loosening and tooth fractures; in the control group, interim complete dentures caused discomfort [14]. The success rate for the immediately loaded prostheses was 100%. Mean bone losses of 0.61 ± 0.21 and 0.53 ± 0.18 mm were reported for test and control implants, respectively. No statistically significant differences were found between loading protocols [14].

Finally, in a recent literature review, the same authors stated that immediate loading with FFA prostheses in the upper jaw is associated with successful treatment outcome, if adequate criteria are used to evaluate the patient, choose the implant, and perform the surgical and prosthetic treatment [13]. This is an interesting perspective. In fact, the survival and success of an implant-supported FFA rehabilitation are determined by a number of factors, some related to the patient's general condition (medical status [30, 31], smoking habit [26], and history of periodontal disease [32]), some related to the local patient anatomy and recipient bone site (quantity and quality of bone [6, 9, 11–13]), and others related to the implant system used [13, 21, 27, 33], surgical and prosthetic protocols adopted [6, 11–14, 16, 34, 35], and experience and skills of the surgeon/prosthodontist [13].

Our prospective short-term clinical study seems to confirm the findings reported in the current literature, although the distributions of patients, implants, and prosthetic rehabilitations were peculiar. In fact, the distribution of patients was statistically inhomogeneous with respect to sex (*p* = 0.001), smoking habit (*p* = 0.004), and history of periodontal disease (*p* < 0.0001). The majority of patients (20 versus 4) were in fact female; although smokers were numerically inferior to nonsmokers, the percentage of smokers was rather high (24/115, 20.8%); finally, all patients (100%) had a history of chronic periodontal disease. As is known, smoking and history of periodontal disease are risk factors for implant therapy failure [26, 28, 30, 32]; nevertheless, in this clinical trial, implant survival was high (95.9%, patient-based), and we did not report failures caused by infection or peri-implantitis, with a relatively low incidence of biological complications (4.1% patient-based, with a reversible peri-implant mucositis affecting 2 fixtures in a single patient). Peri-implant marginal bone resorption was quite low, with overall values of 0.21 mm (±0.15 mm; median, 0.18 mm; 95% CI, 0.19–0.23 mm) and 0.25 mm (±0.11; median 0.25 mm; 95% CI, 0.24–0.26 mm), respectively, 1 and 3 years after implantation, in line with the current literature [11–14, 23]. With respect to implant site, in our study the majority of the fixtures were placed in the maxilla (130), against a smaller number of mandibular implants (85), with a nonhomogeneous distribution (*p* = 0.002); overall, the distribution of implants was also inhomogeneous by location (*p* < 0.0001), with a majority of fixtures placed in the posterior region (138/215, 64.2%). It is well known that bone quality is lower in the posterior areas, particularly in the maxilla, and can represent a risk factor for the short- and long-term success of implants [6, 9, 11–13, 35]. In our study, most of the implants (201/215, 93.4%) were placed in sites with lower-quality bone (type III and IV bone); only 14 were installed in areas of high density (types I and II bone). On the other hand, most of the fixtures (151) were positioned at sites that in some way regenerated, whereas only 64 implants were placed in native bone. Finally, in our clinical work, the vast majority of the fixtures were positioned in fresh extraction sockets (144) rather than in completely healed edentulous ridges (71) (again, there was a statistically significant difference between the two groups, with *p* < 0.0001). Immediate implant placement in fresh postextraction sockets is a well-known and clinically successful procedure [14, 19]; however, the insertion and primary stabilization of the fixture in a postextraction socket may not be simple, and the procedure is certainly more risky than the conventional technique and positioning of the fixture in a fully healed edentulous ridge [14, 19]. Nevertheless, in this clinical work, only 2 implants failed, both in the posterior maxilla of a patient who had been subjected to bone regeneration through sinus lift. The excellent results reported in this study were certainly made possible by the clinical experience of the operator (a single experienced operator performed all surgeries), but also and especially by the use of a tapered implant with peculiar macrotopography, characterized by knife-edge threads, capable of maximizing the primary implant stabilization in difficult situations (as in the case of low-bone-quality sites, regenerated sites, or postextraction sockets) [21–23, 33]. This thread design, may, in fact, result in maximized bone-to-implant contact and compressive force resistance and minimized shear force production; this could help in maintaining implant stability in the immediate postplacement healing period [21–23, 33]. In addition, the implants used in the present study featured a surface with peculiar micro- and nanotopographical characteristics. In fact, the superimposition of an ultrastructural treatment (by means of incorporation of calcium ions) on the classic sandblasted surface made it possible to obtain a nanostructured surface with increased contact area and high surface energy [22, 24]. As reported in the literature, a surface with increased surface area and free energy can stimulate and accelerate the deposition of new bone, thus transforming the primary stabilization of a mechanical nature into a stable biological, secondary stabilization, paramount for the integration, and success of the fixture [15, 17]. Finally, with respect to prosthetic protocols, it should be noted that in the present work almost all FFAs were loaded immediately (27/36, 75%); only 9 were conventionally loaded. Moreover, in most cases, the opposing arch was represented by FFAs or natural teeth, whereas only 2 cases were represented by removable dentures with resin teeth. These elements, again, give value to the implant system employed in this clinical work, which is able to support the load in difficult contexts, as previously reported in the literature [21–23]. It is interesting that, in this study, the number of prosthetic complications was slightly higher than the number of biological complications, with an incidence of 5.5% (2/36 FFAs), considering the definitive prostheses only. Indeed, in 2 cases there was chipping or fracture of the ceramic coating; this against two arches supported by 4 and 6 implants, respectively. In addition, there were problems in the provisionalization period, with 4 fractures of temporary restorations, which were repaired or replaced. Again, these adverse events occurred in FFAs supported by 4 (2 fractures) or 6 (2 fractures) implants. No complications occurred in FFAs supported by 7 or 8 implants, which, in accordance with the literature, suggests that placement of a greater number of implants, in the medium and long term, reduces the incidence of prosthetic complications affecting FFAs [35]. However, in any case, all complications reported in this article were technical in nature and, therefore, affected the prosthetic superstructures; we did not report mechanical complications, that is, adverse events occurring at the implant-abutment connection. The type of connection used in the implant system in this clinical work (a 5 mm deep conical connection combined with an internal hexagon) seems to ensure high mechanical stability, at least in the short term; however, this should be confirmed in the medium and long term.

Globally, the complication-free survival of restorations (88.9%) reported in this study was higher than reported in previous work [10, 16, 29], given the complexity of FFAs (restorations that are, by nature, subject to a higher complication rate than single crowns or fixed partial dentures supported by implants).

However, the present clinical study has limitations. First, the number of patients treated and, consequently, the number of restorations placed are relatively low: further studies are needed on larger numbers of patients to confirm the extremely positive outcomes reported here. Second, this is a short-term study (mean follow-up, 2.0 ± 0.8 years). Therefore, all positive evidence that emerged in this study will have to be verified in the medium and long term, by following these implant-supported prosthetic rehabilitations over time and recording survival and complications. Finally, for proper assessment of the stability of marginal bone tissue around the implants, evaluation through panoramic radiography is definitely a second choice. The panoramic radiographs are subject to more distortion than periapical intraoral x-rays, so the latter would be preferable for a more accurate calculation of bone loss around the implants over time.

## 5. Conclusions

In the present prospective clinical study, FFAs supported by tapered implants with knife-edge thread design and a nanostructured, calcium-incorporated surface were extremely successful in the short term, with high implant survival rates (95.9% patient-based, 99.1% implant-based). The peri-implant marginal bone loss was 0.21 ± 0.15 and 0.25 ± 0.11 mm, respectively, 1 and 3 years after implant placement. A low incidence of biological (4.1% patient-based, 0.9% implant-based, and 2.7% restoration-based) and prosthetic (5.5% restoration-based) complications was reported, for an overall “complication-free” survival of restorations of 88.9%. These positive clinical outcomes need to be confirmed in further long-term studies on larger samples of patients.

## Figures and Tables

**Table 1:** Patient-related information.

	Number of patients	*p* value^*∗*^
Overall	24 (100%)	
*Gender*		
Males	4 (16.7%)	0.001
Females	20 (83.3%)	
*Age at surgery*		
30–39	5 (20.83%)	0.232
40–49	2 (8.33%)	
50–59	9 (37.5%)	
60–69	4 (16.7%)	
≥70	4 (16.7%)	
*Smoking habit*		
Yes	5 (20.8%)	0.004
No	19 (79.2%)	
*History of periodontal disease*		
Yes	24 (100%)	<0.0001
No	0 (0%)	

^*∗*^Chi-square test.

**Table 2:** Implant-related information.

	Number of implants	*p* value^*∗*^
Overall	215 (100%)	
*Site*		
Maxilla	130 (60.5%)	0.002
Mandible	85 (39.5%)	
*Position*		
Incisors	50 (23.3%)	<0.0001
Cuspids	27 (12.6%)	
Premolars	65 (30.2%)	
Molars	73 (33.9%)	
*Surgical protocol*		
Immediate	144 (67%)	<0.0001
Conventional	71 (33%)	
*Bone quality*		
Types I-II bone	14 (6.5%)	<0.0001
Type III bone	143 (66.5%)	
Type IV bone	58 (27%)	
*Bone conditions*		
Grafted sites	151 (70.2%)	<0.0001
Nongrafted sites	64 (29.8%)	
*Final insertion torque*		
<40 N·cm	23 (10.7%)	<0.0001
≥40 N·cm	192 (89.3%)	
*Length*		
7.0 mm	5 (2.3%)	<0.0001
8.5 mm	9 (4.2%)	
10.0 mm	52 (24.2%)	
11.5 mm	73 (33.9%)	
13.0 mm	44 (20.5%)	
15.0 mm	32 (14.9%)	
*Diameter*		
3.5 mm	88 (40.9%)	<0.0001
4.0 mm	92 (42.8%)	
4.5 mm	27 (12.6%)	
5.0 mm	8 (3.7%)	

^*∗*^Chi-square test.

**Table 3:** Prosthesis-related information.

	Number of FFAs	*p* value^*∗*^
Overall	36 (100%)	
*Site*		
Maxilla	21 (58.3%)	0.317
Mandible	15 (41.7%)	
*Opposing dentition*		
FFA (metal-ceramic teeth)	24 (66.7%)	<0.0001
Natural teeth	10 (27.8%)	
Complete removable prosthesis (resin teeth)	2 (5.5%)	
*Loading protocol*		
Immediate loading	27 (75%)	0.002
Conventional loading	9 (25%)	
*Number of implants*		
4 implants FFA	8 (22.2%)	0.0001
6 implants FFA	20 (55.6%)	
7 implants FFA	1 (2.8%)	
8 implants FFA	7 (19.4%)	

^*∗*^Chi-square test.

**Table 4:** Bone loss around the implants at different follow-up controls, in mm (implant-level).

	Baseline- 1 year	Baseline- 3 years
	*n* ^*∗*^; mean (SD); median; CI 95%	*n* ^*∗*^; mean (SD); median; CI 95%
All implants	213; 0.21 (±0.15); 0.18; 0.19–0.23	213; 0.25 (±0.11); 0.25; 0.24–0.26
Immediate postextraction implants	144; 0.20 (±0.14); 0.15; 0.18–0.22	144; 0.23 (±0.12); 0.24; 0.22–0.24
Implants in healed ridges	69; 0.23 (±0.17); 0.2; 0.19–0.27	69; 0.28 (±0.09); 0.29; 0.26–0.3

*n* ^*∗*^ = number of the implants examined.
